# Supplementary material for: Comparative Renal Safety of Tirzepatide and Semaglutide: An FDA Adverse Event Reporting System (FAERS)—Disproportionality Study
Source: J Clin Med. 2025 Oct 29;14(21):7678. doi: 10.3390/jcm14217678 (PMC12610529; doi:10.3390/jcm14217678)
Supplement: Supplementary file 1 [file jcm-14-07678-s001.zip › jcm-3914393-supplementary.pdf]

Table S1- STROBE CHECKLIST

| Item No. | STROBE Item | Explanation |
|---|---|---|
| 1 | Title and abstract | Title specifies study design (“FAERS disproportionality analysis”). Abstract provides background, objectives, methods, results, and conclusions. *(Page 1)* |
| 2 | Background / rationale | Introduction provides scientific background on T2DM, AKI, GLP-1 RAs, and rationale for using pharmacovigilance data. *(Pages 1-2)* |
| 3 | Objectives | Clear statement of primary and secondary objectives at end of Introduction. *(Page 2)* |
| 4 | Study design | Methods section describes a **retrospective cross-sectional pharmacovigilance study** using FAERS data. *(Page 3)* |
| 5 | Setting | Study setting: FAERS database (publicly available U.S. FDA adverse event reporting system) covering reports from January 2022 to September 2025. *(Page 3)* |
| 6 | Participants | All domestic AE reports listing tirzepatide or semaglutide as primary suspect drugs were included. Exclusion: duplicates based on case identifiers. *(Page 3-4)* |
| 7 | Variables | Outcome variable: acute kidney injury (AKI), defined using standardized MedDRA preferred terms. Exposure variables: tirzepatide or semaglutide. Covariates: demographic characteristics and serious outcomes. *(Page 4-6)* |
| 8 | Data sources / measurement | FAERS database, standardized MedDRA coding, 2×2 contingency tables for disproportionality metrics. *(Page 3-4)* |
| 9 | Bias | Potential reporting bias discussed in Discussion section (limitations of spontaneous reporting). *(Page 9)* |
| 10 | Study size | All available reports from Jan 2022–Sep 2025 included; no sample size calculation applicable. *(Page 4)* |
| 11 | Quantitative variables | ROR and PRR calculated; definitions and thresholds described in Methods. *(Page 5)* |
| 12 | Statistical methods | Disproportionality analysis using ROR and PRR; thresholds per Evans et al. 2001; descriptive statistics for demographics and outcomes. *(Page 3)* |
| 13 | Participants (Results) | Total reports identified for tirzepatide and semaglutide, number of AKI cases, sex distribution. *(Page 4-7 )* |
| 14 | Descriptive data | Demographics and outcomes described (Figures 1–3). *(Page 6–7)* |
| 15 | Outcome data | AKI frequencies, disproportionality metrics (ROR, PRR) presented in Table 1. *(Page 6)* |
| 16 | Main results | Comparative ROR and PRR between tirzepatide and semaglutide, year-wise trends, outcomes. *(Pages 4-6)* |
| 17 | Other analyses | Subgroup analyses (serious outcomes) described in Results and Figure 3. *(Page 7)* |
| 18 | Key results | Summarized in Discussion: lower AKI reporting frequency with tirzepatide vs semaglutide. *(Page 7-8)* |
| 19 | Limitations | Reporting bias, under-reporting, lack of causality explicitly discussed in Discussion. *(Page 9)* |
| 20 | Interpretation | Balanced interpretation of findings in light of existing evidence and study limitations. *(Page 8-9)* |
| 21 | Generalizability | External validity discussed (real-world pharmacovigilance, relevance to clinical practice). *(Page 8)* |
| 22 | Funding | “No external funding” disclosed in Funding section. *(Page 10)* |
